# Extracellular pyruvate kinase M2 promotes osteoclastogenesis and is associated with radiographic progression in early rheumatoid arthritis

**DOI:** 10.1038/s41598-022-07667-6

**Published:** 2022-03-07

**Authors:** Dong Woo Han, Yong Seok Choi, Hye Won Kim, Seunghwan Shin, You-Jung Ha, Eun Ha Kang, Jun Won Park, Jin Kyun Park, Kichul Shin, Yeong Wook Song, Yun Jong Lee

**Affiliations:** 1 Department of Translational Medicine, College of Medicine, Seoul National University, Seoul, Korea; 2 Medical Science Research Institute, Seoul National University Bundang Hospital, Seongnam, Korea; 3 Division of General Internal Medicine, Department of Internal Medicine, Seoul National University Bundang Hospital, Seongnam, Korea; 4 Division of Rheumatology, Department of Internal Medicine, Seoul National University Bundang Hospital, 82 Gumi-ro, 173 Beongil, Bundang-gu, Seongnam-si, 13620 Gyeonggi-do Korea; 5 Department of Internal Medicine, Seoul National University Hospital, Seoul, Korea; 6 Department of Internal Medicine, Seoul National University College of Medicine, Seoul, Korea; 7 Division of Rheumatology, Seoul Metropolitan Government-Seoul National University Boramae Medical Centre, Seoul, Korea; 8 WCU Department of Molecular Medicine and Biopharmaceutical Sciences, Medical Research Institute, Seoul National University College of Medicine, Seoul, Korea

**Keywords:** Molecular biology, Molecular medicine, Pathogenesis, Rheumatology

## Abstract

Extracellular PKM2 (exPKM2) levels have been reported to be increased in several cancers and inflammatory diseases, including rheumatoid arthritis (RA). This study aimed to investigate the association of circulating exPKM2 levels with radiographic progression in RA patients and the effect of exPKM2 on osteoclastogenesis. Plasma and synovial fluid exPKM2 levels were significantly elevated in RA patients. Plasma exPKM2 levels were correlated with RA disease activity and were an independent predictor for radiographic progression in RA patients with a disease duration of ≤ 12 months. CD14^+^ monocytes but not RA fibroblast-like synoviocytes secreted PKM2 upon stimulation with inflammatory mediators. Recombinant PKM2 (rPKM2) increased the formation of tartrate-resistant acid phosphatase (TRAP)-positive multinuclear cells and resorption pit in osteoclast precursors, dose-dependently, even in the absence of receptor activator of nuclear factor-kappa B ligand (RANKL). rPKM2 treatment upregulated the expression of dendrocyte-expressed seven transmembrane protein (DC-STAMP) and MMP-9 via the ERK pathway. Although rPKM2 did not directly bind to RAW264.7 cells, extracellular application of pyruvate, the end-product of PKM2, showed effects similar to those seen in rPKM2-induced osteoclastogenesis. These results suggest that exPKM2 is a potential regulator of RA-related joint damage and a novel biomarker for subsequent radiographic progression in patients with early-stage RA.

## Introduction

Chronic hypoxia of the synovium, driven by chronic inflammation and synoviocyte proliferation, promotes synovial inflammation and bone erosion in patients with rheumatoid arthritis (RA)^1,2^. In a hypoxic microenvironment cellular metabolism shifts from oxidative phosphorylation to anaerobic glycolysis, which provides metabolic advantages for cell growth and survival^3^. Indeed, hexokinase-2 (HK-2) and 6-phosphofructo-2-kinase/fructose-2, 6-bisphosphatase 3 (PFKFB3) were markedly upregulated in RA synovial tissue^4–7^. Increased glycolysis is associated with the activation and differentiation of innate and adaptive immune cells in RA^8–10^. Accordingly, inhibition of glycolysis ameliorated tissue-destruction by RA fibroblast-like synoviocytes (FLSs) and arthritis severity in a murine model of inflammatory arthritis^4–7^. Therefore, alterations in the glycolytic pathway might be critical in RA pathogenesis.

Pyruvate kinase (PK) is a key rate-limiting enzyme that converts phosphoenolpyruvate to pyruvate in the final step of glycolysis. Increased expression of PK accelerates glycolysis. Among the four PK isoforms (PKL, PKR, PKM1, and PKM2), PKM2 is abundant in proliferative cells such as stem cells, tumor cells, and stromal cells in chronic inflammatory tissues such as RA joints^11–16^. Interestingly, PKM2 can be released into the extracellular space; PKM2 levels are elevated in the blood or feces of patients with cancer and chronic inflammatory diseases^11,17,18^. Extracellular PKM2 (exPKM2) can induce the proliferation and migration of cancer cells and promote angiogenesis via integrin β1^19–21^. However, the role of exPKM2 in RA pathogenesis has not been fully elucidated. In this study, we investigated the expression levels of PKM2 in the synovial tissue, synovial fluid (SF), and plasma of RA patients. Additionally, we evaluated the clinical implications of plasma exPKM2 levels and the effect of exPKM2 on osteoclastogenesis.

## Methods

### Study subjects

RA was diagnosed according to the 1987 American Rheumatism Association criteria^22^. Synovial tissues were obtained from 15 patients (12 RA and 3 OA patients) undergoing joint-replacement surgery or synovectomy. RA-FLSs were isolated from the synovium of 7 RA patients. The synovial tissues of 5 RA and 3 OA patients were fixed with 4% buffered paraformaldehyde and embedded in paraffin. SF samples were collected from a different group of 25 RA and 5 OA patients.

We consecutively enrolled 139 patients with RA (age 54.3 ± 11.9 years, 120 females) and 47 sex- and age-matched healthy donors (3:1 ratio; age 54.4 ± 12.3 years, 40 females). The demographic and clinical features of the participants are summarized in Supplementary Tables 1–3 (Additional File 1).

### Clinical and radiographic assessment

Clinical and laboratory data were collected at the time of blood sampling. RA disease activity was assessed according to the DAS28-ESR^23^. The patients were categorized based on the RA type as follows: active RA, DAS28-ESR > 3.2; remission, DAS28-ESR < 2.6; and early RA, disease duration ≤ 12 months.

Radiographs of the hands and feet were taken at the time of blood sampling and after a mean of 26.8 ± 16.6 months. Radiographic damage was assessed by two blinded trained investigators (YJH and HWK) using the modified Sharp/van der Heijde score (mSHS)^24^. The interobserver intraclass correlation coefficient (ICC) was 0.979 (95% confidence interval 0.974–0.984), and the smallest detectable change in mSHS was 1.59 during the follow-up period. Erosive disease was defined as an mSHS erosion score ≥ 1 at baseline^25^. Radiographic progression was defined as ΔmSHS ≥ 1 unit/year, while erosive or narrowing disease progression was defined as ∆mSHS ≥ 1 unit/year in the corresponding subscore as previously described^26^.

### Cell lines and reagents

The cell lines and reagents used in this study are described in the Supplementary Methods (Additional File 2). Cryopreserved peripheral blood mononuclear cells (PBMCs) were thawed, and CD3^+^, CD14^+^, and CD19^+^ cells were isolated using a magnetic bead-based method (Miltenyi Biotec, Miltenyi Biotec, Auburn, CA, USA), according to the manufacturer’s instructions.

### Immunohistochemistry and double immunofluorescence (IF) staining

After the paraffin-embedded synovial tissue was deparaffinized, immunohistochemistry analysis of intracellular PKM2 was performed following a previously reported protocol with minor modifications^27^. The sections were stained with anti-PKM2 antibodies (1:1600) for 3 h at room temperature. The number of PKM2-immunostained cells was manually counted among synovial lining and sublining stromal cells. At least 3 high-power fields without an area of lymphoid aggregates were randomly selected, and a minimum of 1000 cells were analyzed on each slide.

Double IF staining was performed by incubating the sections with anti-vimentin (1:100), anti-CD68 (1:100), anti-CD20 (1:400), or anti-CD3 (1:50) antibodies at 4 °C overnight. The slides were incubated with Texas Red-X-conjugated anti-mouse IgG antibody for 1 h at room temperature in the dark. The slides were incubated with the anti-PKM2 antibody (1:100) at 4 °C overnight, followed by incubation with Alexa Fluor^®^-488-conjugated anti-rabbit IgG antibody for 1 h at room temperature in the dark. For negative controls for immunostaining, primary antibodies were omitted and then sections were incubated with corresponding secondary antibodies. PKM2 colocalization with each cell-specific marker was quantitatively analyzed using the Colocalization Threshold plugin of ImageJ Fiji 1.53c software (NIH, Bethesda, Maryland, USA; https://imagej.net/downloads)^28,29^.

### Culture of FLSs, PBMCs, and THP-1 cells

The isolation and culture of FLSs were performed as previously described^27^. Cell proliferation was analyzed by using a 3-(4,5-dimethylthiazol-2-yl)-2,5-diphenyltetrazolium bromide (MTT) assay. The THP-1 cell line was cultured and differentiated into macrophage-like cells as described in the Supplementary Methods (Additional File 2). Isolated CD14^+^ monocytes from human PBMCs were cultured in complete MEM (α-MEM; 10% fetal bovine serum (FBS), 1% penicillin/streptomycin) supplemented with 50 ng/mL M-CSF. CD3^+^ or CD19^+^ lymphocytes were grown in complete RPMI medium (RPMI 1640 medium; 10% FBS, 1% penicillin/streptomycin).

### Osteoclastogenesis and osteoclast activity assay

RAW264.7 cells were seeded in 48-well plates (1 × 10^3^ cells/well) and cultured in α-modified minimum essential medium (MEM) containing 10% FBS in the presence of recombinant PKM2 (rPKM2), pyruvate, or receptor activator of nuclear factor kappa-Β ligand (RANKL). Tartrate-resistant acid phosphatase (TRAP) staining was performed after 5 days, and TRAP-positive multinucleated cells were counted under a light microscope. Osteoclast activity was assessed by measuring the area of resorption pits using a calcium phosphate-coated 24-well plate as previously described^30^. The details of the pit formation assay methods are provided in the Supplementary Methods (Additional File 2). CD14^+^ monocytes were seeded at 1.5 × 10^5^ cells/well in complete MEM, and on the next day, RANKL or rPKM2 was added. The culture medium was replaced every 4 days and multinucleated TRAP-positive cells were counted after 21 days.

### Reverse transcription-polymerase chain reaction (RT-PCR) and immunoblotting

The cellular expression levels of genes and proteins were evaluated by RT-PCR and immunoblotting, respectively, using specific primers and antibodies. The PCR primer pairs are shown in the Supplementary Methods (Additional File 2).

### Measurement of circulating PKM2 and proinflammatory cytokine levels and pyruvate kinase activity

The exPKM2 levels were measured using commercial enzyme-linked immunosorbent assay (ELISA) kits, following the manufacturer’s instructions. The serum levels of TNF-α, IL-6, and VEGF were analyzed with a Luminex 100 system (Luminex, Austin, TX, USA) using a magnetic bead-based immunoassay (R&D Systems, Minneapolis, MN, USA). To evaluate the enzymatic activity of rPKM2, a Pyruvate Assay Kit (Abcam, Cambridge, MA, USA) was used according to the manufacturer’s instructions. All measurements were performed in duplicates.

### Statistical analysis

Data are presented as the mean ± standard deviation or median [interquartile ranges]. Continuous variables were compared using the Mann–Whitney *U* test or Kruskal–Wallis test. Categorical variables were compared using the Chi-square test or Fisher’s exact test as appropriate. Bivariate correlations were analyzed using Spearman’s correlation coefficient. Binary logistic regression analysis (stepwise forward regression) was performed to identify independent variables associated with radiographic progression. P or corrected p values < 0.05 were considered statistically significant. All data were analyzed using SPSS Statistics for Windows version 25 (IBM Corp., Armonk, NY, USA).

### Ethics approval and consent to participate

This study was approved by the Institutional Review Board (IRB No. B-0905/075-013) and was performed according to the recommendations of the Declaration of Helsinki. Informed written consent was obtained from all participants.

## Results

### Increased expression of PKM2 in RA synovial tissues

**Figure 1:**
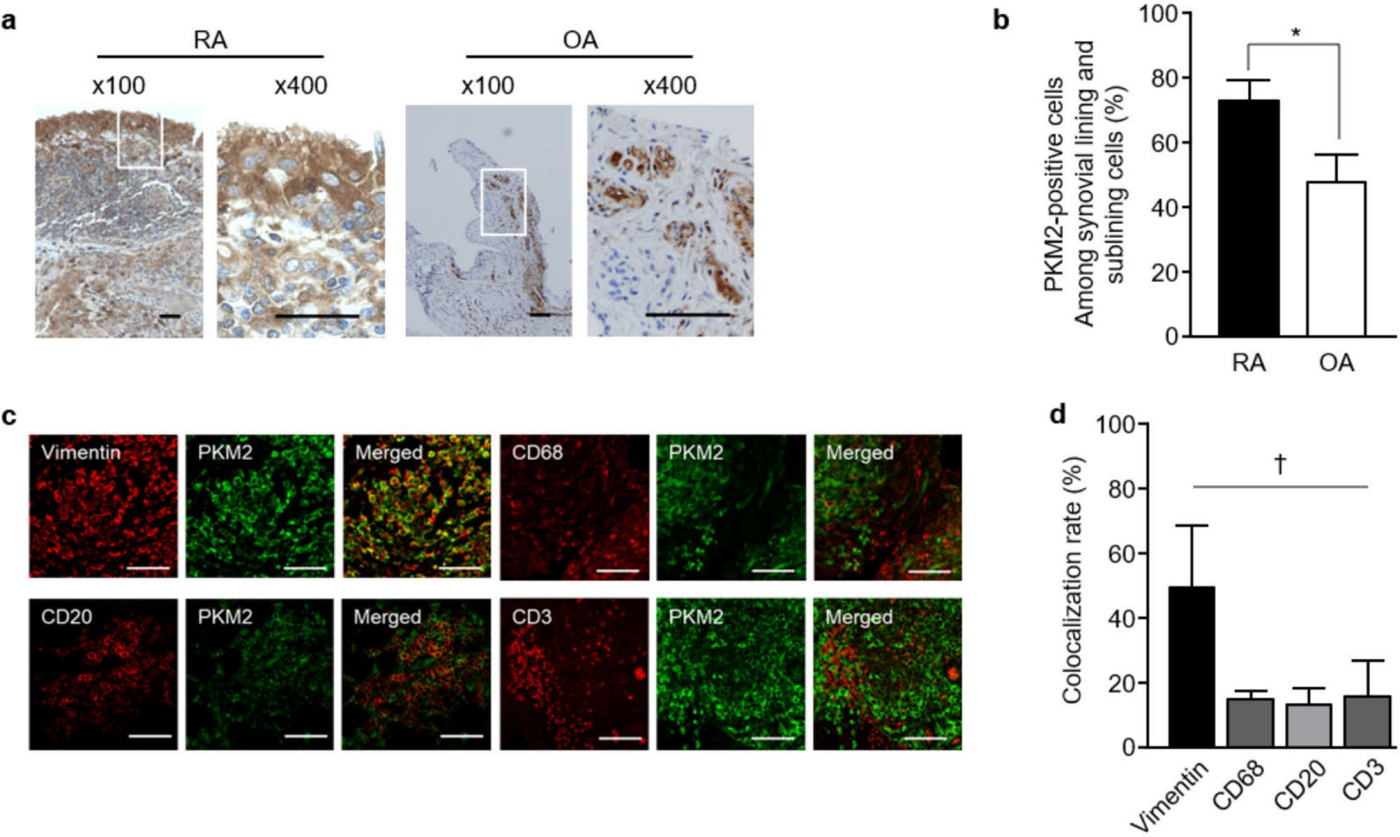
Intracellular PKM2 expression in RA synovial tissues. (**a**) In representative light microscopy photographs, PKM2 immunostaining was increased in RA synovial tissue. White boxes in the left figures (original magnification, × 100) indicate the magnified regions shown in the right figures (× 400). Scale bars = 200 μm. (**b**) The fraction of PKM2-positive cells was significantly higher among synovial lining and stromal cells in RA synovial tissues (n = 5) than in OA (n = 3) synovial tissues. *p = 3.06 × 10^–6^ by the Mann–Whitney *U* test. (**c**) Representative confocal images showing PKM2 (green) expression in vimentin-positive, CD68-positive, CD20-positive, and CD3-positive cells (original magnification, × 400). Scale bars = 100 μm. (**d**) The colocalization rate between PKM2-positive and vimentin-positive cells was significantly higher than that of other cells (n = 4). ^†^p = 0.03 by the Kruskal–Wallis test. Data with error bars were expressed as mean ± SD.

Immunostaining analysis revealed that intracellular PKM2 was expressed in the synovium of RA and osteoarthritis (OA). PKM2-positive stromal cells were distributed in both the lining and sublining layers (Fig. 1). Mononuclear inflammatory cells and vascular endothelial cells also stained positive for PKM2. As expected, the RA synovium had a significantly higher immunosignal intensity (Fig. 1a) and a higher fraction of PKM2-positive stromal cells than the OA synovium (mean 72.9 ± 6.3% vs. 47.5 ± 8.7%, p = 3.06 × 10^–6^; Fig. 1b). To identify PKM2-expressing cells, double IF staining was performed. The colocalization rate of intracellular PKM2 with vimentin (49.3 ± 19.3%) was significantly higher than that with CD68 (15.0 ± 2.5%), CD20 (13.3 ± 5.1%) or CD3 (15.8 ± 11.0%; p = 0.03 by the Kruskal–Wallis test; Fig. 1c,d).

### Upregulated exPKM2 levels in SF samples from RA patients

**Figure 2:**
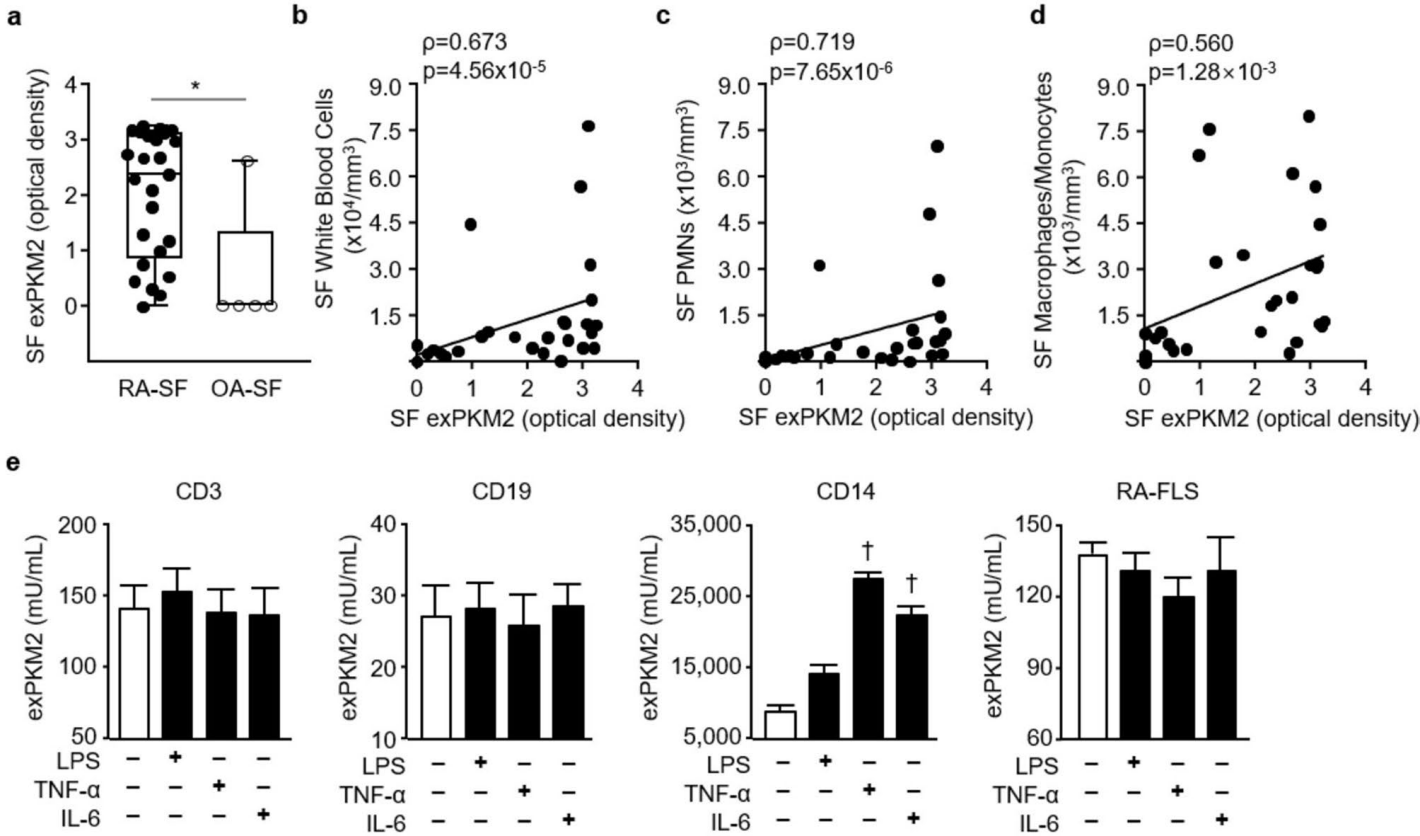
Extracellular PKM2 (exPKM2) levels. (**a**–**d**) In synovial fluid (SF), exPKM2 levels were significantly elevated in patients with RA (n = 25) than those with OA (n = 5) using dimer-specific (**a**; *p = 0.006 by the Mann–Whitney *U* test) ELISA. Data were plotted as box-and-whisker plots. exPKM2 levels were significantly positively related with the counts of SF inflammatory cells; total white blood cells (**b**), polymorphonuclear neutrophils (PMN, **c**), or macrophages/monocytes (**d**). ρ coefficients were calculated by the Spearman method. (**e**) exPKM2 levels were significantly increased when CD14^+^ peripheral blood monocytes were stimulated with 10 ng/mL of TNF-α or 50 ng/mL of IL-6 for 24 h (n = 4; ^†^p = 0.029 by the Mann–Whitney *U* test), but not RA-FLSs (n = 3). LPS (100 ng/mL) also tended to increase exPKM2 levels from CD14^+^ monocytes in the media.

exPKM2 levels were significantly increased in the SF samples of RA patients compared to those of OA patients by using both dimer-specific (median optical density 2.37 [0.86–3.09] vs. 0.0 [0.0–1.30], p = 0.006 by the Mann–Whitney *U* test; Fig. 2a) and nonspecific ELISA kits (274.1 [116.8–313.2] vs. 360.7 [322.8–618.8] ng/mL, p = 0.044; Supplementary Fig. 1a). exPKM2 levels were positively correlated with the numbers of white blood cells including polymorphs and monocytes/macrophages, in the SF samples (Fig. 2b–d, all p < 0.001).

Although neutrophils have been reported to release exPKM2, we examined which cells were the major source of exPKM2. When CD3^+^, CD14^+^, or CD19^+^ PBMCs and RA-FLSs were stimulated with lipopolysaccharide (LPS), TNF-α, or IL-6, exPKM2 levels were significantly elevated only in the conditioned medium of the CD14^+^ cells (Fig. 2e; p = 0.004). Additionally, THP-1 derived macrophages released PKM2 extracellularly when stimulated with these osteoclastogenic stimuli (Supplementary Fig. 1b).

### Elevated exPKM2 levels in the plasma of RA patients

**Figure 3:**
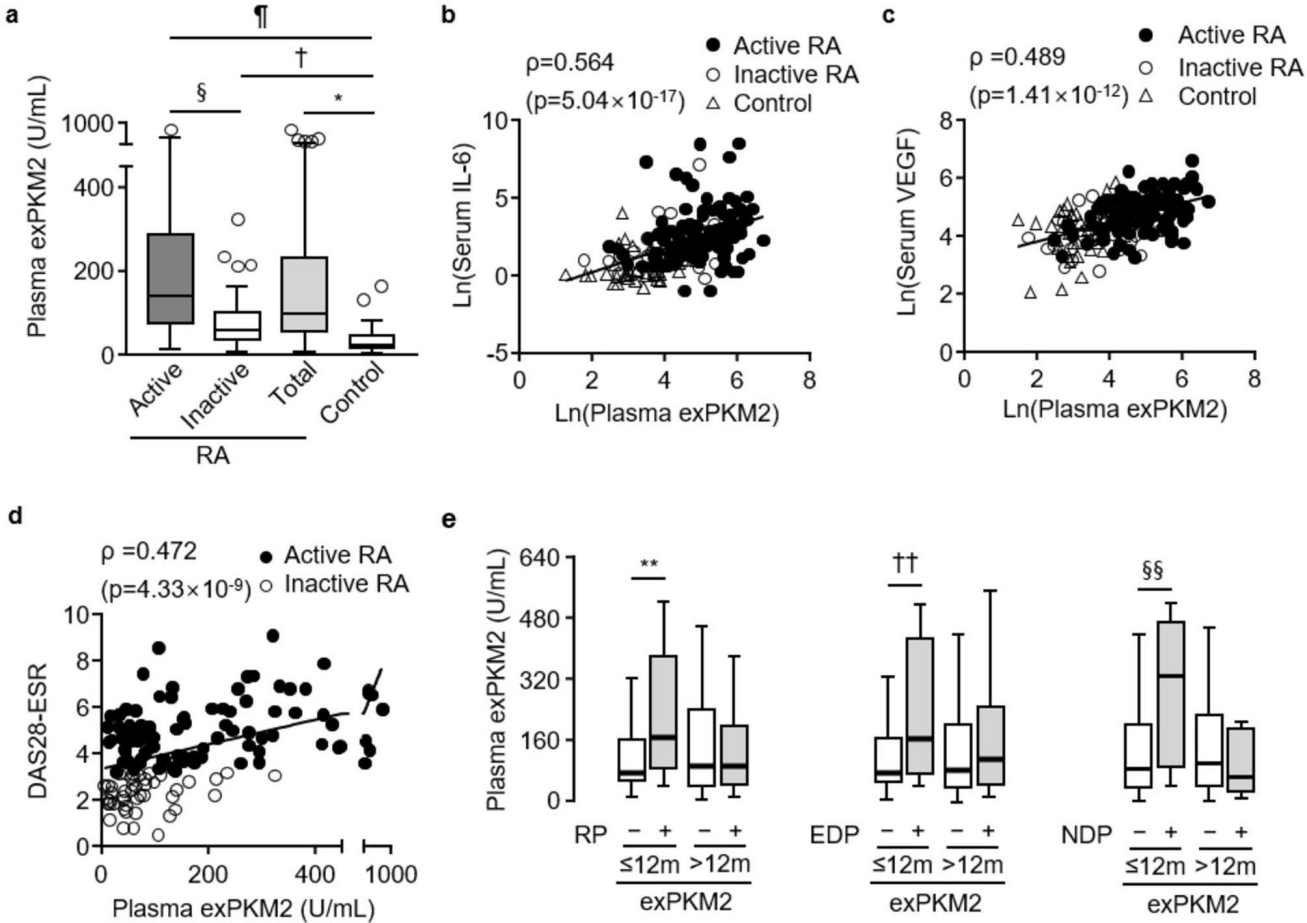
Circulating extracellular PKM2 (exPKM2) levels. levels in RA. (**a**) Plasma exPKM2 levels were significantly increased in total RA group (n = 139) and active (n = 94) or inactive (n = 45) RA subgroups when compared to control (n = 47) group (*p = 6.72 × 10^–13^; ^†^p_c_ = 4.21 × 10^–4^; ^¶^p_c_ = 8.76 × 10^–15^; ^§^p_c_ = 1.71 × 10^–5^ by the Mann–Whitney *U* test). (**b**,**c**) Plasma exPKM2 levels were positively correlated with serum IL-6 and VEGF levels in active and inactive RA patients and controls. (**d**) Plasma exPKM2 levels were significantly correlated with DAS28-ESR in active and inactive RA patients. ρ coefficients were calculated by the Spearman method. (**e**) Among early RA patients (disease duration < 12 months; n = 54), plasma exPKM2 levels were significantly increased in those with radiographic progression (RP), erosive disease progression (EDP), or narrowing disease progression (NDP). Data were plotted as box-and-whisker plots. **p = 0.017; ^††^p = 0.0017; ^§§^p = 0.024 by the Mann–Whitney *U* test.

Then, we examined whether circulating exPKM2 levels were elevated in RA patients and investigated the association of plasma exPKM2 levels with RA disease activity and radiographic progression. Compared to those in the control group (23.7 [16.8–45.4] U/mL), plasma exPKM2 levels were significantly higher in the total RA group (97.7 [55.3–232.3] U/mL) and the active (141.1 [74.2–286.7] U/mL) and inactive (59.3 [36.9–101.6] U/mL) RA subgroups (Fig. 3a, all p < 0.0001). The plasma exPKM2 levels were also positively correlated with the serum IL-6 (p = 5.04 × 10^–17^; Fig. 3b) and VEGF levels (p = 1.41 × 10^–12^; Fig. 3c). In RA patients, exPKM2 levels were significantly correlated with age (p = 0.024), swollen and tender joint counts (both p < 0.001), ESR levels (p = 1.82 × 10^–14^), and CRP levels (p = 3.71 × 10^–9^; Supplementary Table 4). In addition, the exPKM2 levels were significantly correlated with DAS28-ESR scores (p = 4.33 × 10^–9^; Fig. 3d). RA patients taking methotrexate showed significantly lower exPKM2 levels than those not taking methotrexate (p = 0.047; Supplementary Fig. 1c). But this difference did not remain statistically significant when RA patients were stratified into those with active or inactive disease. Additionally, patients with erosive disease had higher exPKM2 levels than those without erosive disease (p = 0.037; Supplementary Fig. 1d). However, exPKM2 levels were not associated with the baseline mSHS scores (Supplementary Table 4).

When RA patients were stratified into radiographic progressors (n = 46) and nonprogressors (n = 80), exPKM2 levels were comparable between the two subgroups (Supplementary Tables 5, 6). However, among patients with early RA (n = 47), radiographic progressors (n = 13) had a significantly higher baseline level of exPKM2 (171.9 [88.5–382.0] vs. 77.8 [55.4–165.4], p = 0.017; Fig. 3e). The exPKM2 levels were also significantly elevated in early RA patients with erosive (p = 0.0017) or joint narrowing disease progression (p = 0.024; Fig. 3e). As expected, when compared to patients with nonearly RA, early RA patients showed a significantly lower prevalence of patients with erosive disease (38.9% vs. 75.3%, p = 1.77 × 10^–5^) and that of patients taking antirheumatic drugs including methotrexate (46.3% vs. 81.2%, p = 1.84 × 10^–5^). Nevertheless, ∆mSHS levels were comparable between early and nonearly RA patients. In logistic regression models for multivariate analysis, the exPKM2 level remained an independent variable associated with radiographic progression in the early RA subgroup (Table 1).

**Table 1:** Factor for radiographic progression in 47 early RA patients.

	Univariate logistic regression		Multivariate logistic regression	
	OR [95% CI]	P value	OR [95% CI]	P value
Age (year)	1.048 [0.987–1.113]	0.124	–	
Sex (female)	0.948 [0.160–5.627]	0.953	–	
Age at onset (year)	1.048 [0.989–1.113]	0.121	–	
Elderly onset^a^	2.031 [0.516–7.992]	0.311	–	
Menopause	3.889 [0.904–16.725]	0.068	–	
Erosive disease	2.139 [0.584–7.829]	0.251	–	
Neutrophil counts	1.000 [1.000–1.001]	**0.033**	–	
Neutrophilia^b^	2.929 [0.365–23.198]	0.313	–	
VEGF	1.003 [0.997–1.009]	0.382	–	
High VEGF^c^	1.067 [0.266–4.281]	0.927	–	
ExPKM2	1.006 [1.001–1.011]	**0.015**	1.007 [1.001–1.012]	**0.012**^d^
High ExPKM2^c^	3.306 [0.839–13.026]	0.087	4.600 [1.038–20.381]	**0.045**^e^

*OR* odds ratio, *CI* confidence interval; ^a^Defined as RA disease onset after the age of 60 years; ^b^Absolute neutrophil counts > 7800/mm^3^; ^c^High exPKM2 or VEGF was defined as the highest quartiles of exPKM2 or VEGF levels in RA patients, respectively; ^d^Continuous variables were entered without transformation or categorization; ^e^Continuous variables were converted to binary categorical variables, except for age. p values <0.05 in univariate analysis in bold.

### exPKM2 promotes osteoclastogenesis via the extracellular signal-regulated kinase (ERK) pathway

**Figure 4:**
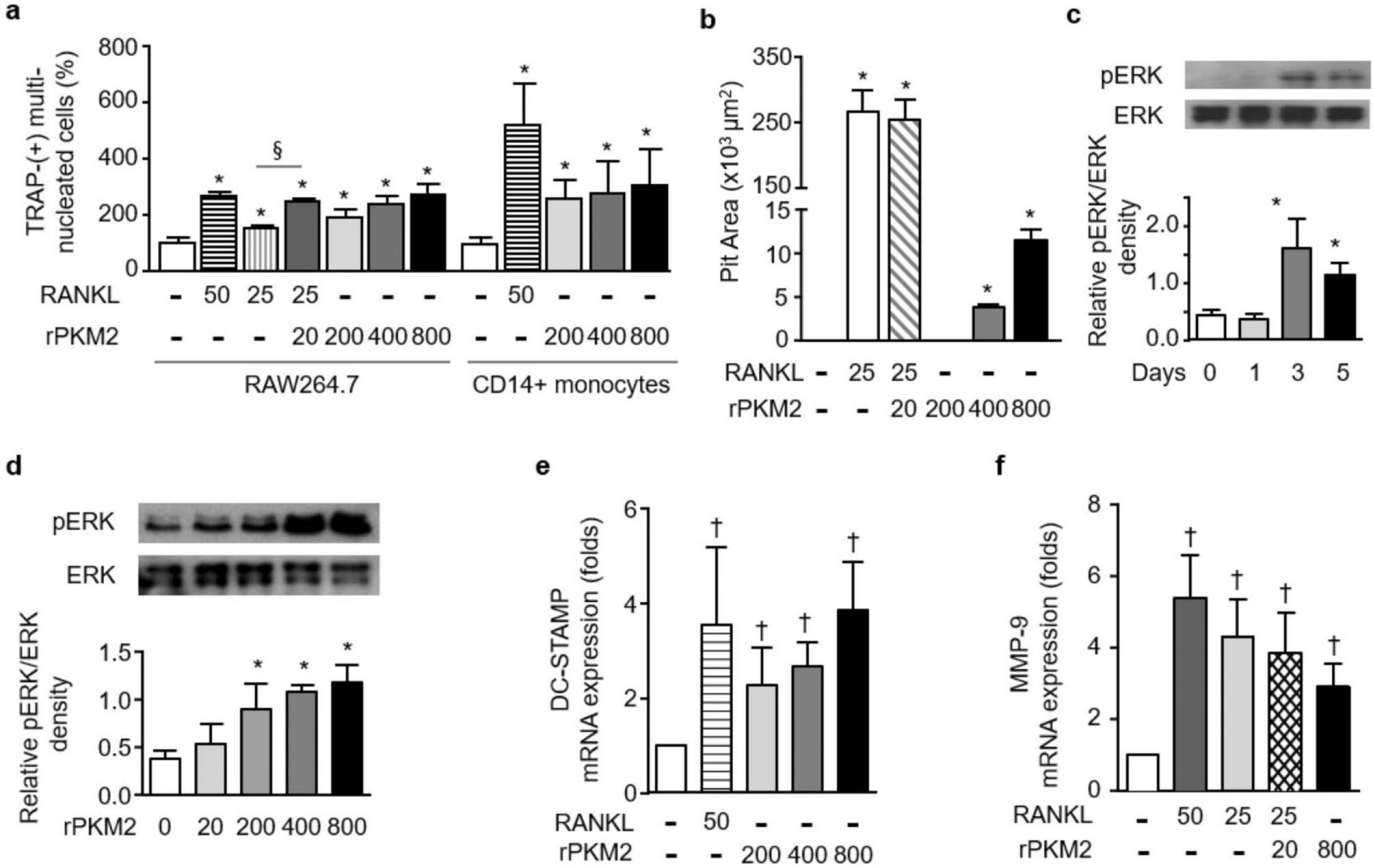
Extracellular PKM2 (exPKM2)-induced osteoclastogenesis through ERK signaling pathway. (**a**) TRAP-positive multinucleated cells were increased when RAW264.7 or human CD14^+^ cells were treated with RANKL or recombinant PKM2 (*p = 0.029 by the Mann–Whitney *U* test versus the unstimulated condition; n = 4). Suboptimal RANKL (25 ng/mL) induced formation of TRAP-positive multinucleated cells was significantly augmented when added a low dose (20 ng/mL) of recombinant PKM2 (rPKM2; §, p = 0.029). (**b**) In osteoclast activity assay, resorption pit area was significantly increased by rPKM2 or RANKL (*p = 0.029 versus the unstimulated condition; n = 4). (**c**,**d**) The level of phospho-ERK was significantly increased in RAW264.7 cells treated with rPKM2 for 3 days and in a dose-dependent mode (*p = 0.029 versus the unstimulated condition; n = 4). Uncropped images are shown in Supplementary Fig. 3a,b. (**e**,**f**) When RAW264.7 cells were treated with RANKL or rPKM2, DC-STAMP and MMP-9 mRNA levels were significantly increased (^†^p = 4.11 × 10^–5^; n = 3). Data with error bars were expressed as mean ± SD.

The exPKM2 levels measured were in the range of 237–883 ng/mL in RA SF samples (Supplementary Fig. 1a). Therefore, RAW264.7 cells or human CD14^+^ cells were treated with rPKM2 at concentrations from 200 to 800 ng/mL to study the effect of exPKM2 on osteoclastogenesis. There was no significant difference in the cellular growth rates of RAW264.7 cells treated with rPKM2 alone (Supplementary Fig. 2a). rPKM2 enhanced the formation of TRAP-positive multinucleated cells from RAW 264.7 cells and CD14^+^ PBMCs in a dose-dependent manner (all p < 0.05, Fig. 4a) even in the absence of RANKL stimulation. Additionally, upon suboptimal stimulation with RANKL (25 ng/mL), treatment with 20 ng/mL rPKM2 significantly increased the number of TRAP-positive multinucleated cells (p = 0.029). Furthermore, rPKM2 significantly increased the resorption pit area (p = 0.029; Fig. 4b), even though the increase in the pit area in the rPKM2 group was smaller than that in the RANKL group.

Phospho-ERK levels were upregulated upon treatment with rPKM2 for 3 days, and the increase was dose dependent (p = 0.029; Fig. 4c,d). However, the expression levels of nuclear factor of activated T cells 1 (NFATc1) and p38 mitogen-activated protein (MAP) kinase were not significantly affected by rPKM2 treatment for 5 days (Supplementary Fig. 2c). Treatment with rPKM2 significantly increased dendrocyte-expressed seven transmembrane protein (DC-STAMP) and MMP-9 transcript levels (both p < 0.0001; Fig. 4e,f). Moreover, treatment with U0126, an ERK inhibitor, reversed the rPKM2-induced upregulation of DC-STAMP (Supplementary Fig. 2d).

### Extracellular pyruvate promotes osteoclastogenesis

**Figure 5:**
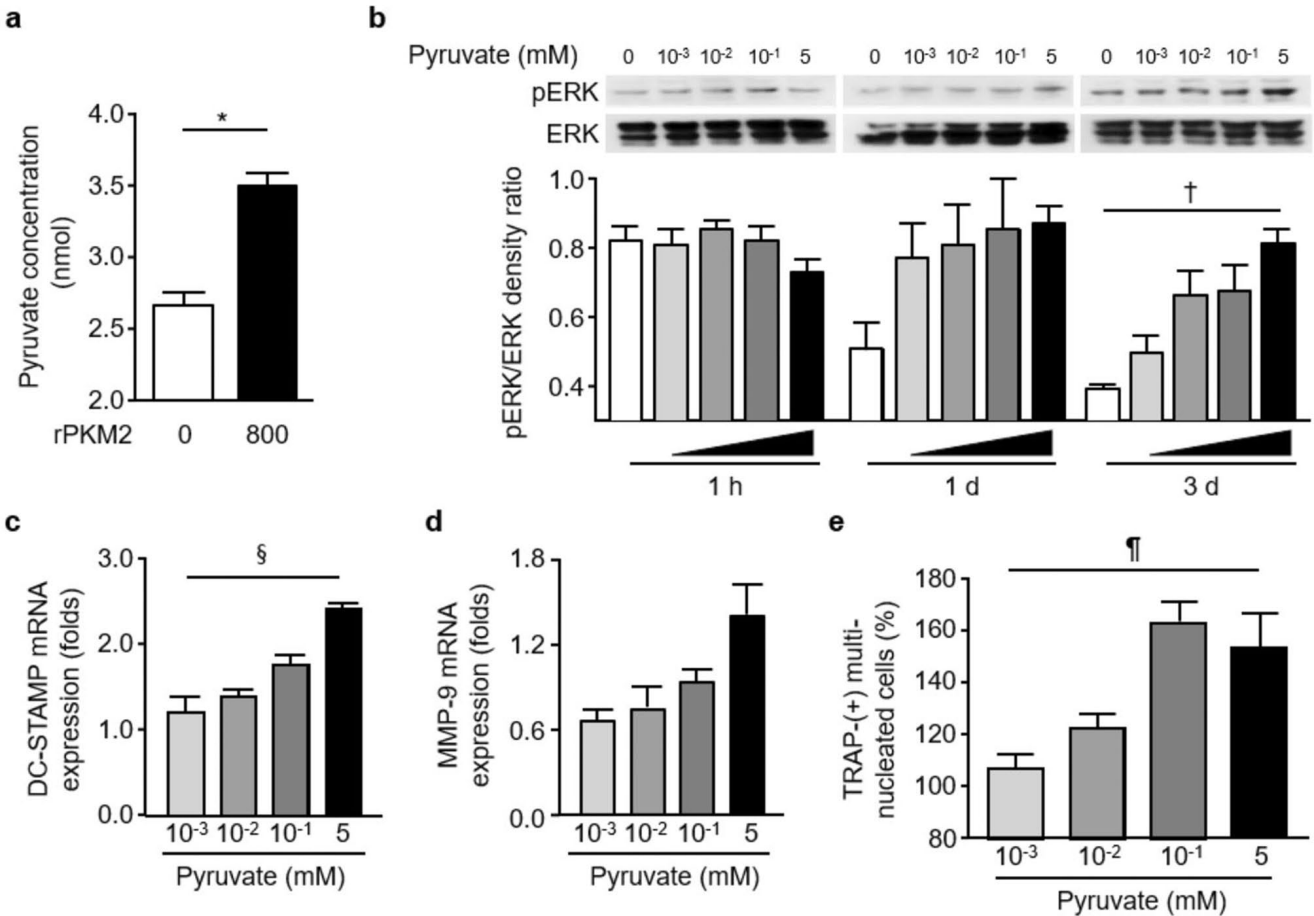
Effects of extracellular pyruvate on osteoclastogenesis through ERK signaling pathway. (**a**) recombinant PKM2 (rPKM2) catalyzed the conversion of phosphoenolpyruvate to pyruvate. Pyruvate increased additionally by an average 31.5% in 60 min with 800 ng/mL of rPKM2 (*p = 0.029 by the Mann–Whitney *U* test; n = 4). (**b**) With pyruvate treatment (0.001–5 mM), the level of phospho-ERK was significantly increased in RAW264.7 cells in a dose-dependent mode (^†^p = 0.007 by Kruskal–Wallis test; n = 4). Uncropped images are shown in Supplementary Fig. 3c. (**c**,**d**) When RAW264.7 cells were treated with pyruvate, DC-STAMP and MMP-9 mRNA levels were increased (^§^p = 0.005; n = 3). (**e**) Extracellular pyruvate dose-dependently augmented the formation of TRAP-positive multinucleated cells even without RANKL (^¶^p = 0.0012). Data with error bars were expressed as mean ± SD.

Reportedly, exPKM2 can activate the epidermal growth factor receptor (EGFR) signaling pathway in cancer cells^19,20^, and EGFR ligands, including EGF and transforming growth factor-α, can enhance bone resorption^31^. However, flow cytometry analysis revealed that rPKM2 did not directly bind the RAW264.7 cell surface and that RAW264.7 cells did not express EGFR (data not shown). It was reported that the addition of 1–2 mM pyruvate significantly increased RANKL-induced osteoclastogenesis in RAW264.7 cells and murine bone marrow cells^32^. Because rPKM2 was able to actively convert phosphoenolpyruvate to pyruvate (Fig. 5a), we investigated whether pyruvate can also induce the expression of phospho-ERK and DC-STAMP even without RANKL, as exPKM2 could. When RAW264.7 cells were stimulated with 0.001 to 5 mM pyruvate, pyruvate significantly increased the levels of phospho-ERK after 72 h in a dose-dependent manner (p = 0.007, Fig. 5b). Additionally, pyruvate treatment upregulated the expression of DC-STAMP and MMP-9 transcripts in RAW264.7 cells (Fig. 5c,d). Conclusively, similar to exPKM2, 0.001 to 0.1 mM pyruvate significantly augmented the formation of TRAP-positive multinucleated cells (p = 0.0012 by Kruskal–Wallis test, Fig. 5e).

## Discussion

In this study, PKM2 was upregulated in the synovial fluid, plasma and synovial tissues of RA patients. Additionally, exPKM2 was released mainly from activated macrophages and induced osteoclastogenesis via the ERK pathway in a dose-dependent manner, even in the absence of RANKL. Increased plasma PKM2 levels were associated with higher disease activity and radiographic progression, especially in early RA. Overall, exPKM2 reflects the inflammatory burden and actively contributes to RA-related joint damage.

Cytosolic PKM2 is known to be upregulated during cellular growth and to be involved in metabolic reprogramming in various malignancies. Cytosolic PKM2 plays a pivotal role in glycolysis via reversible conversion between the tetramer and dimer forms. Tetrameric PKM2 catalyzes PEP to pyruvate with the production of ATP, whereas dimeric PKM2 has low catalytic activity and increases the production of glycolytic intermediates shunted into the pentose phosphate pathway and nucleotide synthesis. In addition, nonmetabolic functions of cytosolic PKM2 are involved in cancer cell growth/survival, stemness, metastasis, or angiogenesis^11^. Dimeric cytosolic PKM2 can be translocated to the nucleus. Nuclear PKM2 can regulate the transcription of genes targeted by hypoxia-inducible factor 1 (HIF-1) or nuclear EGFR^11^. Furthermore, nuclear PKM2 was reported to regulate the production of TNF-α, IL-1β, MMP-2, and MMP-9 in colon cancer cells and to activate inflammasomes^29,33^. Based on these findings, the function or regulation of intracellular PKM2 has been widely researched and has been an attractive target for cancer therapy.

Interestingly, it has been reported that PKM2 is released from cancer cells. This exPKM2 facilitates tumor angiogenesis, cancer cell migration via the phosphoinositide-3-kinase/protein kinase B (PI3K/Akt) and Wnt/β-catenin pathways, and proliferation via EGFR activation^20,34^. Indeed, exPKM2 was detected in serum or stool samples from cancer patients^19^. Moreover, exPKM2 levels were reported to be elevated in inflammatory disorders such as Crohn’s disease or RA^12,14,16,17^. The present study also revealed that exPKM2 levels were significantly elevated in the joint fluid and blood of RA patients.

In the study cohort, plasma exPKM2 levels were significantly increased and correlated with disease activity indices in RA patients. Strikingly, the baseline exPKM2 levels were associated with radiographic progression in the early RA patients (Table 1) but not in the total RA patients. The significant association of exPKM2 with radiographic progression only in early RA patients may be related to a ceiling effect in radiographic progression^35^ or result from therapeutic intervention. The early RA subgroup included significantly more patients not taking antirheumatic drugs than the nonearly RA subgroup at baseline; antirheumatic drugs could negatively affect RA disease activity and subsequent radiographic progression. Regardless of the causes behind the above findings, the association between exPKM2 levels and radiographic progression in the present study suggests that exPKM2 is a novel biomarker predicting subsequent radiographic progression in early RA patients and high exPKM2 levels underlines the need to control RA activity aggressively and tightly.

Initially, we expected that exPKM2 might be released from activated RA-FLSs, which exhibit the characteristics of cancer cells, including high metabolic activity, migration and invasion into surrounding tissue, since vimentin-expressing RA-FLSs showed the highest PKM2 expression (Fig. 1). However, monocytes/macrophages secreted more PKM2 than RA-FLSs or lymphocytes. The cellular source of exPKM2 could explain the significant correlation between exPKM2 and the number of monocytes/macrophages in SF. Moreover, since osteoclasts are derived from the monocyte/macrophage lineage and osteoclast generation is critical for joint destruction, we studied the autocrine or paracrine effect of exPKM2 on osteoclastogenesis. We observed for the first time that extracellular rPKM2 increased formation of TRAP-positive multinucleated cells from human CD14^+^ monocytes and RAW264.7 cells in a dose-dependent manner. Interestingly, the formation of TRAP-positive multinucleated cells was also enhanced upon treatment with a low dose of rPKM2 in the presence of suboptimal RANKL concentrations. However, rPKM2-induced osteoclasts exhibited lower bone resorptive activity than RANKL-induced osteoclasts, suggesting that exPKM2 alone cannot induce fully functional mature osteoclasts. In the present study, rPKM2 upregulated the ERK-dependent expression of DC-STAMP in osteoclast progenitors, and the ERK inhibitor U0126 inhibited rPKM2-induced osteoclastogenesis. DC-STAMP plays an essential role in the fusion of mononuclear osteoclasts, and it can induce the expression of TRAP^36^. The ERK pathway also mediates RANKL- and GM-CSF-induced fusion of osteoclast precursors and DC-STAMP expression^37,38^.

rPKM2 increases phospho-EGFR levels in breast cancer cells^20^, and EGFR increases osteoclast differentiation and survival through its interaction with RANKL^39^. However, we did not observe that nonactivated RAW264.7 cells expressed EGFR or that rPKM2 directly bound to their cell surface. Furthermore, the enhanced levels of phospho-ERK after 3 days of stimulation with rPKM2 indicated that exPKM2 could not act directly through a cell surface receptor. PKM2 catalyzes the final glycolytic step, where PEP is dephosphorylated into pyruvate. Pyruvate can be present in the synovial fluid; pyruvate was detected at concentrations from 0.13 to 0.28 mM in four RA patients in the study of Treuhaft et al.^1^. Additionally, a recent study showed that PEP can be detected in the circulation, and its levels are significantly higher in early RA patients with a therapeutic response to methotrexate^40^. Furthermore, extracellular pyruvate is reported to augment RANKL-induced osteoclastogenesis^32^. Based on the above findings, we investigated the effect of extracellular pyruvate on osteoclastogenesis. The present study finally revealed that the effect of extracellular pyruvate on osteoclastogenesis is in line with that of PKM2. Therefore, exPKM2 secreted from activated monocytes/macrophages can enhance RANKL-independent osteoclastogenesis via catalytic conversion of extracellular PEP to pyruvate.

Although our study provides a novel role of exPKM2 in RA, this study has several limitations. First, plasma PKM2 levels as a prognostic biomarker should be confirmed in large studies. Second, the precise mechanism by which exPKM2 activates and alters intracellular signaling needs further investigation. Concerning the effect of extracellular pyruvate, the clinical implication of PEP or pyruvate levels also needs further study. Finally, therapeutic inhibition of exPKM2 needs to be tested in an in vivo model of inflammatory arthritis.

## Conclusions

exPKM2 levels are increased in the circulation and synovial fluid of RA patients. They are positively associated with RA disease activity and are a significant independent risk factor for radiographic progression in early RA patients. exPMK2 can promote osteoclast differentiation in the early phase via extracellular pyruvate formation and the ERK signaling pathway. Therefore, exPMK2 could be a potential player in RA-related joint damage and a novel biomarker for subsequent radiographic progression in early RA patients.

## Supplementary Information

Supplementary Information 1.

Supplementary Information 2.

## Data Availability

All data generated or analyzed during this study are included in this published article and supplementary information files.
