# Author Correction: Extracellular pyruvate kinase M2 promotes osteoclastogenesis and is associated with radiographic progression in early rheumatoid arthritis

**DOI:** 10.1038/s41598-023-29747-x

**Published:** 2023-02-16

**Authors:** Dong Woo Han, Yong Seok Choi, Hye Won Kim, Seunghwan Shin, You-Jung Ha, Eun Ha Kang, Jun Won Park, Jin Kyun Park, Kichul Shin, Yeong Wook Song, Yun Jong Lee

**Affiliations:** 1 Department of Translational Medicine, College of Medicine, Seoul National University, Seoul, Korea; 2 Medical Science Research Institute, Seoul National University Bundang Hospital, Seongnam, Korea; 3 Division of General Internal Medicine, Department of Internal Medicine, Seoul National University Bundang Hospital, Seongnam, Korea; 4 Division of Rheumatology, Department of Internal Medicine, Seoul National University Bundang Hospital, 82 Gumi-ro, 173 Beongil, Bundang-gu, Seongnam-si, 13620 Gyeonggi-do Korea; 5 Department of Internal Medicine, Seoul National University Hospital, Seoul, Korea; 6 Department of Internal Medicine, Seoul National University College of Medicine, Seoul, Korea; 7 Division of Rheumatology, Seoul Metropolitan Government-Seoul National University Boramae Medical Centre, Seoul, Korea; 8 WCU Department of Molecular Medicine and Biopharmaceutical Sciences, Medical Research Institute, Seoul National University College of Medicine, Seoul, Korea

Correction to: *Scientific Reports* 10.1038/s41598-022-07667-6, published online 07 March 2022

The original version of this Article contained an error in the Funding section.

“This work was partially supported by Basic Science Research Program through the National Research Foundation of Korea (NRF) funded by the Ministry of Education (grant number 2015R1D1A1A01060468, to Lee YJ), by a grant from the Ministry of Science, ICT and Future Planning (grant number NRF-2015M3A9B6052011, to Song YW), and by Chong Kun Dang pharmaceutical Corp. (grant number 800-20180006, to Lee YJ).”

now reads:

“This work was partially supported by Basic Science Research Program through the National Research Foundation of Korea (NRF) funded by the Ministry of Education (grant number 2015R1D1A1A01060468, to Lee YJ), by a grant from the Ministry of Science, ICT and Future Planning, Republic of Korea (grant number NRF-2015M3A9B6052011 and NRF-2020M3E5E2037430, to Song YW), and by Chong Kun Dang pharmaceutical Corp. (grant number 800-20180006, to Lee YJ).”

The original Article has been corrected.

