# Supplementary material for: Extracellular pyruvate kinase M2 promotes osteoclastogenesis and is associated with radiographic progression in early rheumatoid arthritis
Source: Sci Rep. 2022 Mar 7;12:4024. doi: 10.1038/s41598-022-07667-6 (PMC8901694; doi:10.1038/s41598-022-07667-6)
Supplement: Supplementary file 1 — Supplementary Information 1. [file 41598_2022_7667_MOESM1_ESM.docx]

**Supplementary tables and figures**

**Supplementary Table 1.** Baseline characteristics of study participants who provided blood samples

|  | RA (n=139) | Controls (n=47) |
| --- | --- | --- |
| Age (years) | 54.3±11.9 | 54.4±12.3 |
| Female | 120 (86.3%) | 40 (85.1%) |
| Smoker | 21 (15.1%) |  |
| Disease duration (months) | 61.4±75.2 |  |
| Early RA | 54 (38.9%) |  |
| WBC (mm^3^) | 7324.8±2223.8 | 5843.0±1670.8 |
| ESR (mm/h) | 25.4±25.1 | 10.5±8.7 |
| CRP (mg/dL) | 1.4±2.3 | 0.1±0.1 |
| DAS28-ESR | 4.2±1.7 |  |
| Active disease | 94 (67.6%) |  |
| DAS28-CRP | 3.9±1.5 |  |
| Total mSHS (0-448) | 3.0±6.5 |  |
| Erosion subscore | 1.6±3.4 |  |
| Joint space narrowing subscore | 1.4±3.8 |  |
| Erosive disease | 85 (61.2%) |  |
| Seropositivity | 130 (93.5%) |  |
| RF/CCP | 116 (83.5%)/119 (87.5%) |  |
| Medications |  |  |
| MTX | 76 (54.7%) |  |
| DMARDs | 94 (67.6%) |  |
| Glucocorticoid | 64 (46.0%) |  |
| Comorbidities |  |  |
| Osteoporosis | 25 (18.5%) |  |
| Hypertension | 34 (24.5%) |  |
| Diabetes mellitus | 10 (7.19%) |  |
| Dyslipidemia | 9 (6.47%) |  |
| Chronic hepatitis B | 5 (3.62%) |  |
|  | | |

Values are expressed as numbers (percentages) and mean ±SD. Early RA was as denoted to RA with disease duration ≤ 12 months. WBC, white blood cells; ESR, erythrocyte sedimentation rate; CRP, C-reactive protein; DAS28, disease activity score in 28 joints; mSHS, modified Sharp van der Heijde score; RF, rheumatoid factor; anti-CCP, anti-cyclic citrullinated peptide antibody; MTX, methotrexate; DMARDs, disease-modifying anti-rheumatic drugs.

**Supplementary Table 2.** Characteristics of study participants who provided synovial fluid (SF) samples

|  | RA (n=25) | OA (n=5) | p value |
| --- | --- | --- | --- |
| Female | 23 (92.0%) | 5 (100%) |  |
| Age (years) | 57.9±14.0 | 59.0±3.3 |  |
| Disease duration (years) | 9.1±8.5 | - |  |
| ESR (mm/h) | 42.5±26.8 | - |  |
| CRP (mg/dL) | 3.02±3.98 | - |  |
| DAS28-ESR | 3.87±1.19 | - |  |
| Medications |  |  |  |
| MTX | 17 (68.0%) | - |  |
| Other synthetic DMARDs | 14 (56.0%) | - |  |
| Biologic DMARDs | 4 (16.0%) | - |  |
| Glucocorticoids | 13 (52.0%) | - |  |
| SF White blood cells (/mm^3^) | 14766 [4250~12850] | 232 [170~290] | 0.0005 |
| SF PMNs (/mm^3^) | 10670 [1758~9708] | 1 [0~3] | 0.0005 |
| SF Macrophage/monocytes (/mm^3^) | 2803 [943~3986] | 163 [77~255] | 0.0005 |
| SF Lymphocytes (/mm^3^) | 1507 [499~1940] | 66 [29~96] | 0.0014 |

All SF samples were collected from the knee joints. Values are expressed as numbers (percentages) and mean ±SD or median [25~75 percentiles] according to the distribution. RA, rheumatoid arthritis; OA, osteoarthritis; ESR, erythrocyte sedimentation rate; CRP, C-reactive protein; DAS28, disease activity score in 28 joints; MTX, methotrexate; DMARDs, disease-modifying antirheumatic drugs; PMN, Polymorphonuclear neutrophil. P values were calculated by the Mann–Whitney U test.

**Supplementary Table 3.** Characteristics of study participants who provided synovial tissue

|  | RA (n=12) | OA (n=3) |
| --- | --- | --- |
| Female | 11 (100%) | 3 (100%) |
| Age | 53.5±14.8 | 73.3±4.6 |
| Disease duration (years) | 8.4±6.5 | - |
| ESR (mm/h) | 32.5±28.8 | - |
| CRP (mg/dL) | 2.04±2.31 | - |
| DAS28-ESR | 3.59±1.29 | - |
| Medications |  |  |
| MTX | 9 (75.0%) | - |
| Other synthetic DMARDs | 8 (66.7%) | - |
| Biologic DMARDs | 2 (16.7%) | - |
| Glucocorticoids | 7 (58.3%) | - |
| Operation sites | Knee, 4; elbow, 4; hip, 2; wrist, 2 | Knee, 3 |

Values are expressed as numbers (percentages) and mean ±SD; RA, rheumatoid arthritis; OA, osteoarthritis; ESR, erythrocyte sedimentation rate; CRP, C-reactive protein; DAS28, disease activity score in 28 joints; MTX, methotrexate; DMARDs, disease-modifying anti-rheumatic drugs.

| **Supplementary Table 4.** Bivariate Spearman correlation between extracellular PKM2 (exPKM2), pro-inflammatory cytokines, and clinical variables in patients with RA | | | | |
| --- | --- | --- | --- | --- |
| Variables | IL-6 | TNF-α | VEGF | exPKM2 |
| Age | NS | NS | NS | 0.192  (p=0.024) |
| Disease duration | -0.228  (p=0.007) | NS | -0.247  (p=0.003) | NS |
| Swollen joint count | 0.333  (p=6.08×10^-5^) | NS | 0.213  (p=0.012) | 0.347  (p=2.82×10^-5^) |
| Tender joint count | 0.369  (p=7.60×10^-6^) | NS | 0.229  (p=0.007) | 0.297  (p=3.81×10^-4^) |
| DAS28-ESR | 0.467  (p=6.79×10^-9^) | NS | 0.333  (p=6.03×10^-5^) | 0.472  (p=4.33×10^-9^) |
| DAS28-CRP | 0.447  (p=3.45×10^-8^) | NS | 0.269  (p=0.001) | 0.401  (p=9.85×10^-7^) |
| WBC | 0.367  (p=8.58×10^-6^) | NS | 0.236  (p=0.005) | 0.361  (p=1.25×10^-5^) |
| Neutrophils | 0.285  (p=0.001) | -0.258  (p=0.002) | 0.244  (p=0.004) | 0.334  (p=5.97×10^-5^) |
| Lymphocytes | NS | -0.236  (p=0.005) | NS | NS |
| Monocytes | 0.228  (p=0.007) | -0.189  (p=0.025) | NS | 0.188  (p=0.027) |
| Hemoglobin | -0.195  (p=0.021) | NS | -0.171  (p=0.045) | -0.321  (p=1.19×10^-4^) |
| Platelet | 0.275  (p=0.001) | NS | 0.277  (p=0.001) | 0.301  (p=3.10×10^-4^) |
| ESR | 0.472  (p=4.61×10^-9^) | NS | 0.366  (p=9.48×10^-6^) | 0.591  (p=1.82×10^-14^) |
| CRP | 0.497  (p=5.09×10^-10^) | NS | 0.261  (p=0.002) | 0.474  (p=3.71×10^-9^) |
| mSHS total score | NS | NS | NS | NS |
| Joint  narrowing score | NS | NS | NS | NS |
| Erosion score | NS | NS | NS | NS |
| Data are presented as Spearman coefficient ρ (p value). NS, not significant. DAS28, disease activity score in 28 joints; WBC, white blood cells; ESR, erythrocyte sedimentation rate; CRP, C-reactive protein; mSHS, modified Sharp van der Heijde score. | | | | |

**Supplementary Table 5.** Radiographic damage scores and the proportion of radiographic progressor in RA patients (n=126)

|  | At baseline | Follow-up |
| --- | --- | --- |
| Total mSHS score | 5.0 [0-25.3] | 7.0 [1-31.3] |
| Erosion subcore | 2.0 [0-12.5] | 4.0 [0-15.3] |
| Joint space narrowing subscore | 2.0 [0-10.0] | 3.5 [0-13.3] |
| Radiographic progression |  | |
| Total | 46 (36.5%) | |
| Erosion | 30 (23.8%) | |
| Narrowing | 25 (19.8%) | |

Values are expressed as numbers (percentages) or median [25~75 percentiles]. mSHS, modified Sharp/van der Heijde score. Radiographic progression was defined as ΔmSHS ≥1 unit/year while erosive or narrowing disease progression was defined as ∆ ≥1 unit/year in the corresponding subscore.

| **Supplementary Table 6.** Clinical features of RA patients with radiographic progression versus those without progression | | | |
| --- | --- | --- | --- |
| Variables | Non-progressor (n=80) | Progressors (n=46) | p value |
| Age | 51.5 [43~61] | 60.5 [46.8~67] | 0.007 |
| Elderly onset RA | 14 (17.5%) | 14 (30.4%) | 0.093 |
| Menopause | 31/70 (44.3%) | 29/41 (70.7%) | 0.007 |
| Disease duration | 24 [6.0~83.5] | 56.1 [8.8~131.0] | 0.043 |
| Early RA | 34 (42.5%) | 13 (26.3%) | 0.112 |
| Swollen joint count | 2.0 [0.0~5.8] | 3.0 [0.0~6.0] | 0.861 |
| Tender joint count | 2.0 [0.0~5.0] | 2.0 [0.0~7.3] | 0.905 |
| PG-VAS | 70 [41~90] | 70 [50~90] | 0.811 |
| ESR (mm/h) | 17.5 [8.3~34.3] | 16.5 [6.8~29.5] | 0.723 |
| CRP (mg/dL) | 0.50 [0.12~1.40] | 0.67 [0.11~2.18] | 0.586 |
| DAS28-ESR | 4.10 [2.80~5.22] | 4.10 [2.86~4.83] | 0.982 |
| DAS28-CRP | 3.66 [2.62~4.79] | 3.79 [2.79~4.66] | 0.700 |
| Active RA | 53 (66.2%) | 32 (69.6%) | 0.702 |
| WBC (/mm^3^) | 6,945 [5,750~8,553] | 7,580 [6,200~9,040] | 0.157 |
| Neutrophils (/mm^3^) | 4,446 [3,460~5,604] | 5,505 [3,996~6,513] | 0.024 |
| Monocytes (/mm^3^) | 465.5 [379.5~640.5] | 600.5 [403.3~729.3] | 0.100 |
| Hb (g/dL) | 12.8 [11.9~13.6] | 12.6 [11.6~13.7] | 0.229 |
| Platelet (x10^3^/mm^3^) | 280 [229~337] | 261 [226~345] | 0.740 |
| IL-6 (pg/mL) | 6.8 [3.2~18.3] | 6.1 [2.1~50.4] | 0.804 |
| TNF-α (pg/mL) | 2.5 [0.5~3.9] | 1.8 [0.0~3.9] | 0.304 |
| VEGF (pg/mL) | 96.7 [60.6~150.9] | 136.0 [83.4~189.2] | 0.019 |
| PKM2 (U/mL) | 86.5 [47.7~213.3] | 124.1 [56.9~255.8] | 0.184 |
| Baseline mSHS scores | 2.0 [0.0~17.8] | 10.0 [3.5~33.0] | 4.79×10^-4^ |
| Erosive RA | 43 (53.8%) | 35 (75.1%) | 0.013 |
| Annual change in mSHS scores | 0.0 [0.0~0.0] | 2.2 [1.4~3.5] | 1.51×10^-21^ |
| PD dose (mg/day) | 0.0 [0.0~0.0] | 2.2 [1.4~3.5] | 0.037 |
| Hypertension | 13 (16.2%) | 16 (34.8%) | 0.017 |
| Osteoporosis | 10 (12.5%) | 13 (28.3%) | 0.027 |

Values are expressed as numbers (percentages) or median [25~75 percentiles] according to the distribution. PG-VAS, Patient global visual analogue scale; ESR, erythrocyte sedimentation rate; CRP, C-reactive protein; DAS28, disease activity score in 28 joints; WBC, White blood cells; Hb, hemoglobin; mSHS, modified Sharp van der Heijde score; PD, prednisolone; NS, not significant. In case of continuous variables, p values were calculated by the Mann–Whitney U test. For nominal data, Chi-squared test was done to calculate p values.

**Supplementary Figures and Legends**

**
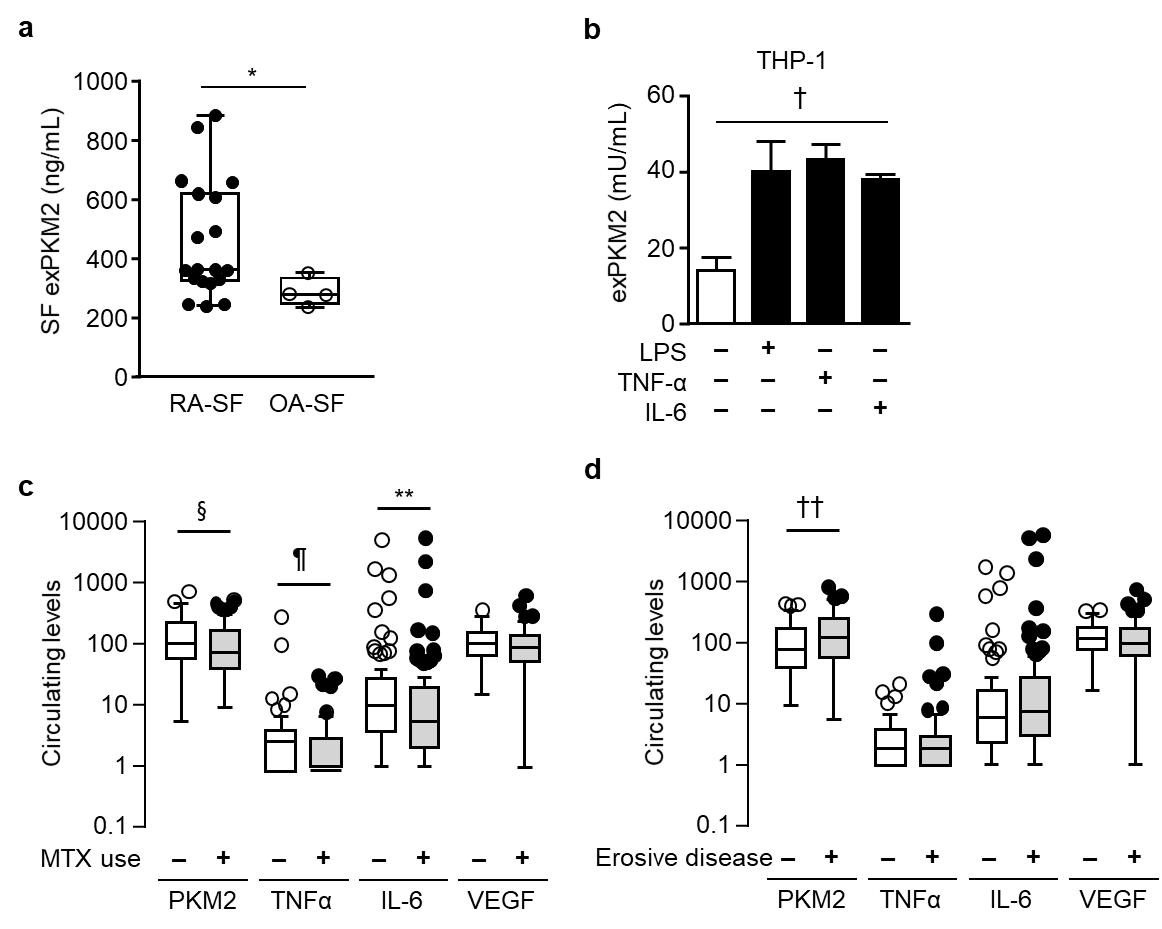
**

**Supplementary Figure 1.** Extracellular PKM2 (exPKM2) levels. **a**. In synovial fluid (SF), exPKM2 levels were significantly elevated in RA patients (n=25) than in OA patients (n=5) using dimer-non-specific ELISA (**a**; *, p=0.044 by the Mann–Whitney U test). Data were plotted as box-and-whisker plots. **b**. exPKM2 levels were significantly increased when THP-1 derived macrophages were stimulated with 100 ng/mL of LPS, 10 ng/mL of TNF-α, or 50 ng/mL of IL-6 for 24 h (n=4; †, p=0.003 by the Kruskal–Wallis test). **c-d**. RA patients taking methotrexate (MTX) had a significantly lower circulating levels of exPKM2 (§, p=0.047 by the Mann-Whitney U test), TNF-α (¶, p=0.012) or IL-6 (**, p=0.020) than those not taking MTX. Additionally, plasma exPKM2 levels were lower in RA patients with erosive disease than in those without erosive disease (††, p=0.037).

**
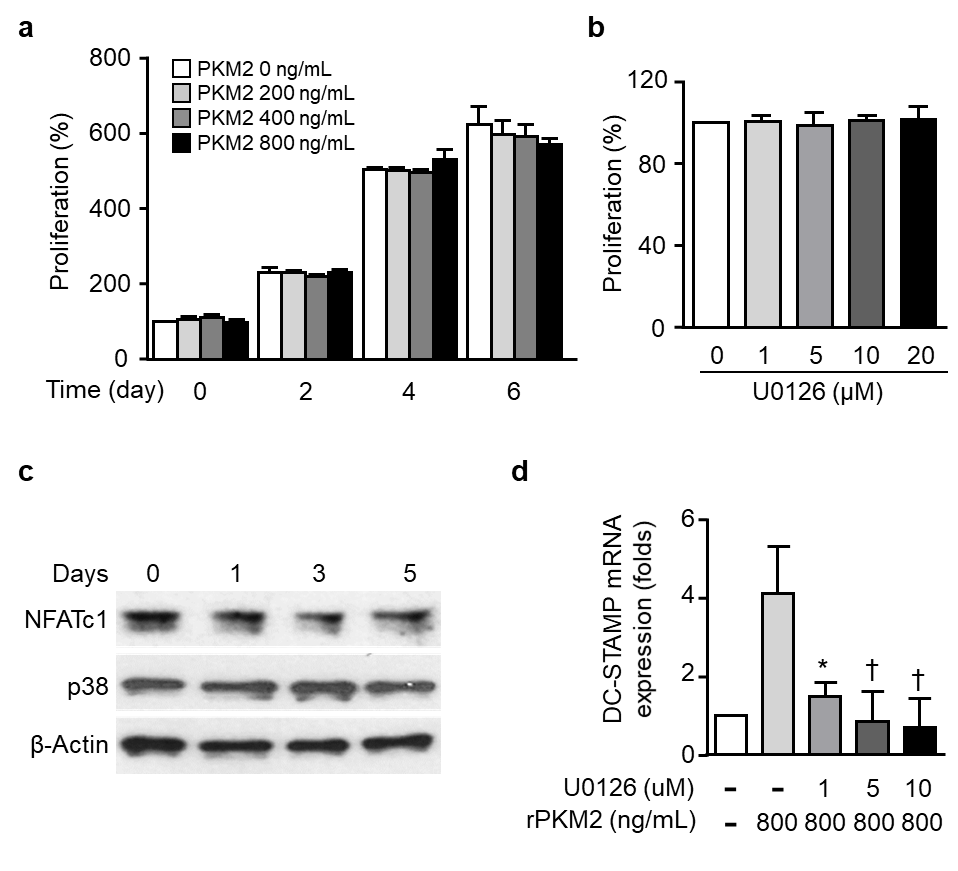
**

**Supplementary Figure 2.** Effects of recombinant PKM2 (rPKM2) or an ERK inhibitor, U0126 in RAW264.7 cells. **a and b.** Using MTT assay, the viability or cellular proliferation did not significantly change after treated with rPKM2 (a) or U0126 (b). **c.** rPKM2 did not affect the expression of NFATc1 and p38 MAP kinase in RAW264.7 cells. Uncropped images are shown in Supplementary Fig. 3d. **d.** rPKM2 induced dendrocyte-expressed seven transmembrane protein (DC-STAMP) mRNA expression was significantly suppressed with 1 to 10 μM of U0126. *, p=0.019 versus the condition without U0126 by the Mann-Whitney U test; †, p=2.88×10^-4^.


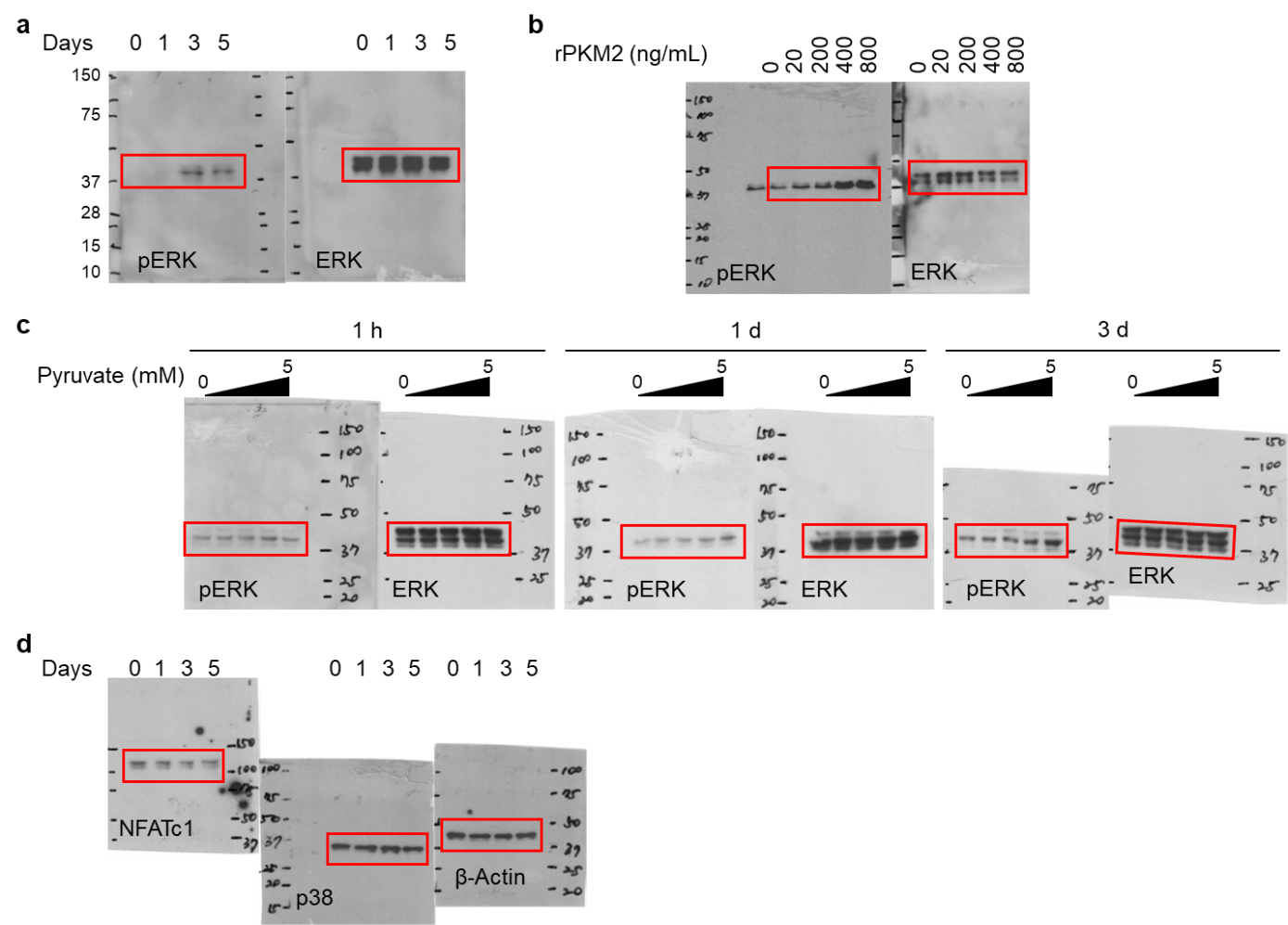


**Supplementary Figure 3.** Uncropped western blot images from Figures 4c (a), Figure 4d (b), Figure 5b (c), and Supplementary Figure 2c (d). The red boxes indicate the cropped regions used in the representative figures.
