# Supplementary material for: Extracellular pyruvate kinase M2 promotes osteoclastogenesis and is associated with radiographic progression in early rheumatoid arthritis
Source: Sci Rep. 2022 Mar 7;12:4024. doi: 10.1038/s41598-022-07667-6 (PMC8901694; doi:10.1038/s41598-022-07667-6)
Supplement: Supplementary file 2 — Supplementary Information 2. [file 41598_2022_7667_MOESM2_ESM.docx]

**Supplementary Materials and Methods**

**Cell lines and reagents**

A murine macrophage cell line RAW264.7, a human monocytic leukemia cell line THP-1, and a human lung cancer cell lines H3122 were bought from American Type Culture Collection (ATCC; Rockville, MD, USA). Human peripheral blood mononuclear cells (PBMC) from 4 anonymous donors were obtained from Koma Biotech (Seoul, Korea). Dulbecco's modified Eagle's medium (DMEM) and minimum essential medium Eagle-alpha modification (α-MEM) were purchased from Welgene (Daegu, Korea). Fetal bovine serum (FBS) was from Atlas Biologicals (Fort Collins, CO, USA) and penicillin-streptomycin and sodium pyruvate were from Gibco (Carlsbad, CA, USA). Phosphoenolpyruvate (PEP) was purchased from Roche Applied Science (Indianapolis, IN, USA). CD14 MACS^®^ MicroBeads were from Miltenyi Biotec Inc (Auburn, CA, USA).

Anti-PKM2 and anti-vimentin antibodies were purchased from Cell Signaling Technology (Danvers, MA, USA) and Leica biosystems (Nussloch, Germany), respectively. Anti-CD68, anti-CD3, and Texas Red-X conjugated secondary antibodies were obtained from Thermo Scientific (Waltham, Massachusetts, USA) while anti-CD20 antibody was purchased from Dako (Glostrup, Denmark). Antibodies to ERK, phospho-ERK, p38 mitogen-activated protein kinase, and phospho-p38 kinase were purchased from Cell Signaling Technology. Anti-EGF receptor and anti-NFATc1 antibodies was ABcam (Cambridge, UK). Phycoerythrin (PE)-conjugated anti-Histidine (His) tag and anti-β-actin antibodies were from Enogene Biotech (New York, NY, USA) and BioLegend (San Diego, CA, USA), respectively. Dimeric PKM2 enzyme-linked immunosorbent assay (ELISA) and total PKM2 ELISA kits were obtained from Schebo Biotech (Netanyastrasse, Giessen, Germany) and Elabscience (Huston, TX, USA), respectively. A pyruvate kinase activity assay kit was obtained from Abcam. Tartrate-resistant acid phosphatase (TRAP) staining and bone resorption assay kits were purchased from Takara (Shiga, Japan) and Cosmo Bio (Tokyo, Japan), respectively.

His-tagged recombinant PKM2 was purchased from Biovision (Milpitas, CA, USA). Recombinant TNF-α, IL-6, M-CSF, and soluble RANK ligand (RANKL) were purchased from Peprotech (Rocky Hill, NJ, USA). Lipopolysaccharide (LPS) and pemetrexed was obtained from Sigma Aldrich (St. Louis, MO, USA). U0126, an ERK inhibitor, was obtained from Promega (Madison, WI, USA)..

**Measurement of extracellular PKM2 (exPKM2)**

The levels of exPKM2 in the plasma and synovial fluid (SF) samples were analyzed using the commercial ELISA kits from Schebo Biotech, following the manufacturer’s instruction with modification. Briefly, test samples (diluted at 1:200 for plasma of control subjects, at 1:500 for plasma from patients with rheumatoid arthritis (RA), and at 1:5000 for SF samples) were added to the 96-well plates precoated with anti-PKM2 antibody. After incubated at 4℃ overnight and then washed repeatedly, the plates were incubated with a biotin-conjugated anti-PKM2 monoclonal antibody for 30 min at room temperature. exPKM2 in the samples was detected using a streptavidin-coupled horseradish peroxidase reaction. The absorbances of the samples were measured at 450 nm using a spectrophotometer (Molecular Devices, San Jose, California, USA). The levels of exPKM2 released from the stimulated RA fibroblast-like synoviocytes (FLSs), CD3, CD14 or CD19-positive PBMCs, and THP-1 cells were analyzed using the ELISA kits. The levels of exPKM2 in the SF samples (diluted at 1:100) was analyzed using the ELISA from Elabscience, to determine the concentration of recombinant PKM2 (rPKM2) used for osteoclast differentiation. All measurements were performed in duplicates.

**Culture and differentiation of THP-1 cell line**

The THP-1 cell line was cultured in RPMI-1640 containing 10% FBS and differentiated into macrophage-like cells by stimulating with phorbol 12-myristate 13-acetate (PMA) for 24 h. The suspended THP-1 cells were transformed to adherent cells after the incubation with PMA. These macrophages were incubated in RPMI-1640 containing 10% FBS without PMA for another 24 h for cell recovery and used for further experiments.

**Pit formation assay**

Osteoclast activity was assayed by measuring the area of resorption pits using calcium phosphate-coated 48-well plate, following the manufacturer’s instructions. The plates were washed once with α-MEM containing 10% FBS. Next, the RAW264.7 cells were seeded onto the plate (2.5🞩10^3^ cells/well). On the next day, the cells were treated with recombinant PKM2 (rPKM2, 0 to 800 ng/mL) alone or in combination with RANKL (0~25 ng/mL) in α-MEM containing 10% FBS. On day 3, the medium was replaced with fresh medium containing rPKM2 alone or a combination of rPKM2 and RANKL. The pit area was evaluated on day 6. Sodium hydrochlorite (5%) was added to the wells to remove the RAW264.7 cells and then the wells were washed with water. The wells were air-dried and the images of all fields were captured under a microscope. The resorbed pit area per well was measured using ImageJ software (NIH, Bethesda, MD, USA).

**Immunoblotting**

The RAW264.7 cells were seeded on the 6-well plate (3.0🞩10^5^ cells/well). On the next day, the cells were cultured in α-MEM containing 10% FBS in the presence of PKM2 (0 to 800 ng/mL). On days 0, 1, 3, and 5, the total cell lysates were obtained using cold RIPA buffer (25 mM Tris-HCl pH 7.6, 150 mM NaCl, 1% NP-40, 1% sodium deoxycholate, 0.1% sodium dodecyl sulfate (SDS)). The total cell lysates were separated on 10% SDS-polyacrylamide gel electrophoresis (SDS-PAGE), and transferred onto a polyvinylidene diﬂuoride (PVDF) membranes. The signals were developed using an enhanced chemiluminescence system (Amersham Biosciences, Little Chalfont, UK). β-actin was used as an internal control. The relative expression of each protein was determined by densitometric analysis using ImageJ software.

**Reverse transcription polymerase chain reaction (RT-PCR)**

The expression levels of target mRNA were analyzed using SYBR green dye. cDNA and target specific primers were added to the power SYBR green PCR master mix (Applied Biosystems, Foster City, CA, USA) and the mixture was subjected to PCR amplification. The PCR conditions were as follows; 1 cycle of 50°C for 2 min, 1 cycle of 95°C for 10 min, followed by 40 cycles of 95°C for 15 s and 60°C for 1 min. The specific murine primers are summarized in the following table.

| Target gene | GenBank accession number | Primer sequences | Reference |
| --- | --- | --- | --- |
| 18S ribosomal RNA | NR_003278 | Forward, 5`-GCAATTATTCCCCATG-AACG-3`  Reverse, 5`-AAGGAGCTTCGCATGC-AGGT-3` | [S1] |
| DC-STAMP | NM_029422 | Forward, 5`-TGCCAGGGCTGGAAGT-TCAC-3`  Reverse, 5`-AAGGAGCTTCGCATGC-AGGT-3` |  |
| MMP-9 | NM_013599 | Forward, 5`-CTGGCAGAGGCATAC-TTGT-3`  Reverse, 5`-CTTCTGAAGCATCAGC-AAAGC-3` |  |

**Fluorescence-activated cell sorting analysis (FACS) of exPKM2 binding**

The RAW264.7 cells were suspended in FBS-free medium (5🞩10^5^ cells/mL) and 5🞩10^5^ cells were transferred into a 15 mL tube. The cells were incubated with His-tagged rPKM2 for 4 h at 37℃ and then washed twice with cell staining buffer (BioLegend). Next, the RAW264.7 cells were stained with PE-conjugated anti-His tag or PE-conjugated mouse IgG2a (κ isotype, BioLegend) antibodies for 20 min on ice in the dark. After washed with cell staining buffer, the RAW264.7 cells were analyzed immediately by BD FACSCalibur^TM^ flow cytometer using CellQuest Pro Software V6 Becton Dickinson, Franklin Lakes, NJ, USA).

**Pyruvate kinase activity assay kit**

To confirm the enzymatic activity of rPKM2, 800 ng of rPKM2 was incubated with 1X kinase buffer (Cell Signaling Technology) for 1 h at room temperature. The mixture was successively kept at room temperature for 1 h after adding 2 mM of PEP. Finally, the level of pyruvate was measured using the colorimetric pyruvate kinase assay kit according to the manufacturer's instructions. PEP in 1X kinase buffer, without rPKM2, served as controls.

**References**

S1. Ray, S. *et al.* Context-dependent function of regulatory elements and a switch in chromatin occupancy between GATA3 and GATA2 regulate Gata2 transcription during trophoblast differentiation. *J. Biol. Chem*. **284**, 4978-88. (2009).
